# Support staff liaising effectively with family caregivers: Findings from a co-design event and recommendation for a staff training resource

**DOI:** 10.3389/fpsyt.2022.977442

**Published:** 2022-09-14

**Authors:** Shoumitro Deb, Bharati Limbu

**Affiliations:** Department of Brain Sciences, Faculty of Medicine, Imperial College London, London, United Kingdom

**Keywords:** experience based co-design, co-production, people with intellectual disabilities, behaviors that challenge, psychotropic medications, psycho-education programme, interdisciplinary collaboration, effective liaison with family caregivers

## Abstract

A high proportion of people with intellectual disabilities (ID) and autism spectrum disorder (ASD) are prescribed psychotropic medications such as antipsychotics, antidepressants etc., outside their licensed indications, primarily for the management of behaviors that challenge (BtC) in the absence of a psychiatric disorder. Examples of BtC are aggression to people and property or self-injury. BtC could be challenging to manage and may cause the person with ID/ASD and their caregivers distress, breakdown of community placement leading to hospitalization, and restrictive practices such as restraint or inappropriate medication use. Caregivers play a pivotal role in the prescribing process. However, many family caregivers feel that they have not been fully involved in the shared decision-making process about the care planning of their relatives with ID/ASD. To address the public health concern regarding the overuse of off-license prescribing in people with ID/ASD, we have recently developed a training programme called SPECTROM (Short-term Psycho-Education for Carers To Reduce OverMedication of people with intellectual disabilities) for direct care staff who support people with ID/ASD within community settings. We used co-production and a modified Experience-Based Co-Design (EBCD) method to develop SPECTROM, which involved a literature review, four focus groups and a co-design event day involving 26 stakeholders. Recommendations from the co-design event day were analyzed by a Programme Development Group (PDG) consisting of 21 stakeholders who made the final recommendations to the project team regarding the contents and the format of SPECTROM, which was finalized after receiving feedback from further 59 stakeholders. SPECTROM has web-based resources introduced through two core modules in face-to-face workshops/training. A small field test found SPECTROM was effective in improving staff's knowledge of psychotropic medications and attitude toward BtC and people with ID (*p* < 0.05). One of the 14 STOMP modules is “Effective liaison with family carers and advocates”. In this paper, we have presented data from the co-design event day recommendations for this particular module. The group recommended ways to improve collaborative working and effective shared decision-making with family caregivers and people with ID/ASD.

## Introduction

Individuals with intellectual disabilities (ID) and autism spectrum disorder (ASD) are at a higher risk of developing behaviors that challenge (BtC) (challenging behaviors) (18–22%) (1, 2). Aggression (about 11%) toward other individuals and objects and self-injurious behavior (SIB) are the most typical forms of BtC (3). BtC poses a significant management problem and is an obstacle to social integration, may lead to caregiver stress, community placement breakdown and hospitalization, and restrictive practices such as physical restraint and inappropriate medication use. BtC also impacts the quality of life (QoL) of individuals with ID/ASD and those who support and care for them (3–8).

Both pharmacological (9) and non-pharmacological psychosocial interventions such as Positive Behavior Support (PBS) (10, 11) are used to manage BtC. A recent meta-analysis found a significant long-lasting moderate overall effect of non-pharmacological interventions on BtC (effect size = 0.573) (12). Other PBS-based non-pharmacological interventions have also been shown to reduce BtC (13) and help with psychotropic medication withdrawal (14). In contrast, the evidence for medication effectiveness in addressing BtC is equivocal (15). Nevertheless, psychotropic medications are prescribed for a large number of people with ID/ASD (49–63%) (16, 17) often to treat BtC outside their licensed indication, such as in the absence of psychiatric disorders (>70%) (17). This is a major public health concern. In the UK, NHS England has embarked on a nationwide campaign called STOMP and STAMP (“STopping Over-Medication of People with learning disabilities, autism or both” and “Supporting Treatment and Appropriate Medication in Paediatrics”) (18).

Family caregivers play an essential part in the care of people with ID/ASD, even when they do not live in the family home. They are the only constant presence in the life of the person with ID/ASD, whereas the professionals and care staff come and go. Therefore, their knowledge of their loved ones is of paramount importance in care provision. On the other hand, caring for relatives with BtC could be stressful, although, notwithstanding that fact, many family caregivers find this caring role fulfilling (19–21). Those family caregivers who support their relatives with ID/ASD at home may sometimes find a conflict between their role as a relative (parents or siblings) and at the same time being a caregiver (22). Caring for a person with ID/ASD who display BtC could be stressful and lead to caregiver burnout, mental and physical health problems, and social isolation (23–25).

Douma et al. (26) asked parents of 745 youths (aged 10-24 years) with moderate to borderline ID, of whom 289 had emotional/behavioral problems of their perception of unmet needs. Most parents (88.2%) needed some support, especially a friendly ear, respite care, child mental health care, and information and these needs were more pronounced for youths with emotional/behavioral problems. The authors recommended that the service providers should provide relevant information to parents including where and how they can access support, activities for the youths, child mental health care and parental counseling. The authors felt that appointment of a case manager could help in these cases. Griffith and Hastings (21) synthesized the qualitative studies on parental views on caring for children and adolescents with ID and elucidated five primary themes; (a) love, (b) altered identity, (c) crisis management, (d) support is not just “challenging behavior” services, and (e) the future: low expectations, high hopes. The authors concluded that support services may cause additional problems and high levels of stress for caregivers, although there were also reports of good practice. From interviews of parents of 48 children with autism, Hastings et al. (19) reported that mothers were found to report both more depression and more positive perceptions than fathers. Regression analyses revealed that paternal stress and positive perceptions were predicted by maternal depression. Maternal stress, on the other hand, was predicted by their children's behavior problems and by their partner's depression.

Two recent surveys of family caregivers' opinions strongly recommended a holistic approach to managing BtC to reduce overreliance on psychotropic medication (27, 28). National and international guidelines recommend non-pharmacological psychosocial interventions as the first line of management option for BtC (29–31). These guidelines and the STOMP and STAMP recommend involving the person with ID/ASD and their families in the decision-making process from the outset. Despite this, family caregivers have reported difficulties accessing pertinent information (32) and partnership working with specialist services (33). This lack of communication and shared decision-making has led to family caregivers' frustration, who expressed their negative feelings by making statements such as “battle” and “banging your head against a brick wall” (34). Some have also complained about being overwhelmed and stressed by bureaucratic processes (35–37).

To address the above issues, we have recently developed online resources accompanied by face-to-face workshop material to train support staff to help reduce the overmedication of people with ID/ASD. The training programme is called SPECTROM (Short-term Psycho-Education for Carers To Reduce Over Medication of people with intellectual disabilities) (https://spectrom.wixsite.com/project) (38). SPECTROM includes internal and external hyperlinked resources with other websites that provide relevant information. There are two core modules, (a) Medication/STOMP and (b) Alternatives to medication, used for face-to-face workshops through which other modules are introduced. There are 12 additional modules. One of these 12 modules is “Effective liaison with families and advocates (39)”. This module was developed to help support staff communicate appropriately with family caregivers and encourage shared decision-making. SPECTROM was created using coproduction and a modified Experience Based Co-design (EBCD) method (40).

EBCD is an interdisciplinary collaborative approach to improving health care services by enabling service-users, caregivers, and professionals to collaborate to co-design better services. EBCD uses service users' experiences as evidence to improve patient experience and health care services. Its crucial feature is equal and close collaboration among all stakeholders, including the patients and their families. It was first piloted in a Head and Neck Cancer service (40). Subsequently, a toolkit was developed by the King's Fund, UK (41). EBCD has the following stages, (a) setting up the project, (b) gathering experiences of patients and staff, (c) co-design events, and (d) reviewing and generating a consensus (40). The co-design method uses participatory experience tools to gather and reflect people's experiences and facilitate quality improvements during the co-design event. Participatory design exercises or tools help to identify “touch points” or critical moments, which are defining moments associated with emotional connections when people come into contact with services (42, 43). This helps to develop or improve services based on experiences (44). For instance, participatory tools such as focus groups or interviews can be carried out to gather participants' experiences and touchpoints of any health care service or intervention. The gathered experiences and touch point will help identify areas of the service or intervention that requires changing or improvement. Through collaboration with patients and staff, methods of improvement are then gathered, and services are improved.

In this paper, we have presented data from the outcome of the codesign event day, specifically the discussion and recommendations for developing the SPECTROM module, “effective liaison with the families and advocates”.

## Materials and methods

SPECTROM was developed according to Medical Research Council's guidelines for creating and evaluating the complex intervention (45). We have described the methodology for developing SPECTROM in detail in a separate paper (38). In brief, information gathered from a literature review, four focus groups and one 1-day co-design event attended by 26 stakeholder representatives were analyzed by a programme development group (PDG) consisting of 21 stakeholders who advised about the content and format of SPECTROM. The stakeholder groups included (a) adults with ID, (b) family caregivers of people with ID, and (c) Community Learning Disability Team (CLDT) members such as social workers, community nurses, behavior therapists, speech and language therapists, occupational therapists, (d) psychiatrists and pharmacists, and (e) general practitioners. The research team developed SPECTROM based on those recommendations, and a draft was sent out to 59 stakeholders for feedback before finalizing the programme. All three stakeholder groups, namely the 26 attendees of the co-design event day, 21 members of the PDG, and 59 participants who participated in the wider stakeholder consultation, were represented by all stakeholder groups. However, the people who represented the stakeholder groups varied from one group to another, with some overlap among the groups.

In this paper, we have presented data from the co-design event day relating to a specific theme, “effective liaison with family carers and advocates”. We have described here the methodology involving the co-design event day.

### Participants in the co-design event

All stakeholders involved in caring for people with ID/ASD were invited to attend a co-design event held on 9th July 2019 at a conference center in London, UK. All correspondence in this study was made by email. Family caregiver organizations in the UK, such as AT-Autism, National Autistic Society (NAS) and Challenging Behavior Foundation (CBF), were asked to send invites to family caregivers on behalf of the SPECTROM project team. Service managers were asked to identify support staff available for the co-design event. We were able to send out 80 invites, and 31 stakeholders confirmed attendance. This included five service managers, five support staff, six trainers, five Community Learning Disability Team (CLDT) members, four psychiatrists, and six family caregivers, including an independent advocate. Eventually, 26 participants attended the event. We divided the participants into five groups, each compromising one service manager, one support staff, one trainer, a CLDT member, a psychiatrist and a family caregiver where possible. The 26 participants who attended the co-design event were representatives of the stakeholder groups of 31 who initially agreed to take part in the event.

The perspective of service users (people with ID/ASD) was gathered *via* the Cornwall Learning Disability Advisory Group (LDAG) in the UK. People with ID/ASD preferred to stay in Cornwall to provide their opinions instead of traveling to London. This group comprised adults with ID/ASD who were prescribed psychotropic medications for BtC or went through psychotropic medication reduction or withdrawal. The authors (SD and BL) formed part of the core team members who floated around all groups to provide support, answer any queries, and encourage equal engagement of participants in each group and interaction and discussion on the day. Other stakeholders, such as general practitioners and pharmacists, were part of the project group and PDG but did not attend the co-design event. Different stakeholders were invited to the co-design event to gain different perspectives. Furthermore, advice on the format and content of SPECTROM training were gathered from PDG separately.

### The development of activities for the co-design event

As part of the SPECTROM study, two sets of focus groups were conducted: one with support staff only (n = 8) and one with service managers and trainers (*n* = 8). The first focus group gathered participants' opinions on psychotropic medication for BtC, views related to BtC, and the relationship between BtC and psychiatric disorders. The second focus group gathered participants' views on the content and the format of SPECTROM training. The focus groups were recorded and analyzed using thematic analysis (46). The themes identified from the focus groups were used as SPECTROM modules and utilized as topics in the co-design event to develop content for SPECTROM modules.

On the co-design event day, participants were asked to prepare content for each topic/theme by completing tasks as activities for the co-design event. The identified topics/themes included (a) autism spectrum disorder (ASD) and attention deficit hyperactivity disorder (ADHD), (b) staff attitude toward the BtC and the person displaying BtC, (c) effective liaison with family caregivers and advocates, (d) effective liaison with health and social care professionals, (e) information for a medication review, (f) communication and interaction issues between caregivers and people with ID/ASD, (g) care/support staff empowerment, (h) communication issues-behavior as a means of communication, (i) effective engagement with people with ID/ASD effectively, and (j) care/support staff reaction to BtC, self-reflection on staff fear/stress, and assessment and management of their own stress.

A guide was developed for each theme to instruct participants on how to complete the activities. The guide informed participants of the allocated theme/topic for discussion, how much time each group had to discuss the issue, provided information on the possible format of the training programme and its target audience, and provided five steps/tasks for the event day. An example of the guide can be found in Supplementary material 1.

### Co-design event

On the day of the co-design event, attendees were registered and given their allocated group numbers. We provided participants handouts on the event's agenda, identified themes from both sets of focus groups, themes for discussion, and guidelines for the day's activities (crib sheet). Additionally, views of people with ID/ASD on BtC and its management based on a Dutch study by Wolkorte et al. were given to provide some suggestions on texts for the contents (see Supplementary material 2) (47). The co-design event started with a PowerPoint presentation for 45 min to provide background information, the aims of the SPECTROM project, the day's structure, and set purposes for the day. Attendees were also shown a video example of a successful antipsychotic withdrawal and its positive effects on the person with ID/ASD. Attendees were given 1 h to work on their activities and 5 min to present their group work. Each group was assigned a flip chart to write their suggestions and recommendations. They were asked to choose a spokesperson to present their work to the whole group. One author (BL) recorded the suggestions and recommendations on a separate paper. The whole event day lasted 6 h, including time for breakfast and lunch. There were two sessions, morning and afternoon, to cover ten potential topics mentioned earlier in the text. Each group received one theme for the morning session and one for the afternoon session. The flip chart papers and any paperwork with suggestions made by the participants were gathered at the end of the event. These and the recorded suggestions were used to develop an action plan for SPECTROM content and format. Attendees were also encouraged to email any comments or further suggestions even after the event. A summary of key points for each theme was developed to generate action plans. The summary points from the co-design event were sent to all members of the PDG for review and feedback. The flip chart papers and notes taken during the presentation of the co-design event day were used to formulate SPECTROM training modules and materials (see Supplementary material 3 for an example of a flip chart). The SPECTROM module was eventually designed based on these recommendations under the guidance of the PDG.

## Results

We have presented the main recommendations from the co-design day that led to the development of the SPECTROM module, “effective liaison with family carers and advocates”. We have presented data under six main themes. We have used an additional heading to cover themes that could not be incorporated into the six main themes.

We have also presented in Box 1 the main headings in the SPECTROM module entitled “Effective liaison with family carers and advocates” derived from the recommendations from the co-design event day. In the actual module, all these main themes were expanded with further detail.

Box 1 **Main headings in the “Effective liaison with family carers and advocates” module in SPECTROM.**

- Understand families' perspectives and show empathy.
- Respect family caregivers' views and be honest with them.
- Communicate with family caregivers effectively and on time.
- Identify a key family member to share information with.
- Let the family know who the key support staff is and how to contact her or him.
- Share information efficiently and openly with family caregivers.
- Where possible and appropriate, involve family caregivers at all stages of decision-making about their loved ones.
- Try to signpost families for help and support.
- If necessary, provide information on independent advocates.
- Encourage family caregivers to train themselves on the management of challenging behavior, medication-related information, non-medicinal management of challenging behavior, STOMP etc., by informing them about the sources for information on these matters.
- Link up the key family caregivers with the key care staff and the key professional.
- Ask family caregivers what information they want about their loved one and how often they want the information.
- Schedule regular meetings with family caregivers as and when appropriate.
- If necessary, link up families together to support each other.

### Information sharing (family knows the person best)

The group recommended that families should receive both good and bad news. Information about their loved ones should be shared in a timely manner and use language understandable to the family caregivers. So, any technical terms/jargon should be avoided. It is desirable to keep a written record of what has been discussed with the family caregivers and share a copy of the document with them for future reference.

Some family caregivers want to be involved in everything and be kept up to date about everything, e.g., what their loved one does during the day (with proof of meaningful activities and opportunities), health and wellbeing concerns, incidents of BtC and how the support staff respond (particularly if as required medication or other more restrictive approaches are used frequently), medical appointments etc. If possible, this should happen, allowing for any reasonable adjustments. It was also highlighted that the family caregivers should be allowed to see their relatives without the presence of the staff if that is their and their relative's preferred option. The staff team should wait for an appropriate moment to discuss issues with the family caregivers.

If the family caregiver has intellectual disabilities, it is essential to find the best way to communicate with them. This may involve providing information in an accessible format, including pictures, sign language, or other communication aids. It is also worth finding out whether they have a communication partner who may help share information appropriately. Staff should not automatically assume that the family caregiver with ID/ASD would be unable to contribute to discussions and decision-making about their relatives because they may not understand what is being said. A referral for an independent advocate for the parent with ID/ASD can be made (with their consent) to support their understanding of a situation and help them make an informed decision and speak up about what they think and want. The group pointed out that not consulting with a family caregiver because they have ID/ASD would breach the UK Equality Legislation (The Equality Act 2010) (48).

It is worth remembering whereas professionals and care staff may come and go, families are the only constant presence in the lives of people with ID/ASD. Families may provide previously unknown information to the staff and other professionals. They may advise on the best way to address their loved one's BtC.

Similarly, family caregivers should be provided with the knowledge that allows early identification of BtC before the behavior in the person with ID/ASD reaches a crisis point and skills to respond to behaviors. This should enable family caregivers to liaise effectively with support staff and inform them about behavior management so that the person's behavior can be managed appropriately at home without medication if possible.

The group recommended that family caregivers be given information on STOMP and STAMP to understand the benefits of medication withdrawal so family caregivers and professionals can liaise effectively. It was suggested that while communicating with family caregivers, any ambiguous terms such as challenging behavior or behaviors that challenge should be defined clearly to avoid any misunderstanding and confusion. The independent advocate in the group recommended that the staff ask family caregivers about the mode and timing of communication, such as through telephone or video calls or face-to-face visits, every week or less often etc. Similarly, if an independent paid advocate is involved, they should discuss a mutually agreed way of communication with the family caregivers.

### Shared decision making

The group raised concern as people with ID and their families are often not involved in decision-making about the person with ID. They are often informed about the decision after that has been made. It was recommended that every effort be made to involve the person with ID/ASD and their families from the outset in any significant decision about the person's care, including health care, management of BtC such as PBS and the use of medication. Family members and the independent advocate in the group highlighted that family caregivers are all different and all have different wishes and expectations of care and support and what they want to be informed about and when. If the person with ID/ASD can tell staff what information should be shared with the family caregivers and in what format, their opinion should be honored.

Family caregivers should be involved in STOMP action plans/medication reviews and psychotropic medication reduction, or withdrawal plans to know which psychotropic medication is being reduced or withdrawn and the side effects associated with the reduction or withdrawal of medications. That way, they will be part of any contingency plan during the withdrawal.

The relevant legal framework should be used if the person with ID/ASD cannot give informed consent about their care. For example, in the UK, the Mental Capacity Act should apply. It is a legal requirement to involve family caregivers in decisions made in their relatives' best interests. The independent advocate in the group reminded people of the importance of the staff team communicating with them appropriately if an independent advocate is appointed to deal with any specific issues. In the UK, when a decision needs to be made about a person needing to move urgently or requiring major medical treatment and family caregivers do not want to be involved in the decision, or there is no family caregiver, or it is inappropriate to consult with family caregivers (for specific reasons), an IMCA (Independent Mental Capacity Advocate) referral may need to be made. When a referral for advocacy is made, the organization employing advocates will inform the staff team about the most appropriate type of advocate.

### Key support staff and key family caregivers

The group recommended that a key person among the staff team be identified. This person should have all the information about the person they support and be the primary source for information sharing. Families should have this person's contact details. Similarly, families should identify a key family member who could work as the family's spokesperson. The staff team and other professionals should have this person's contact details. They could be the first point of contact with the family.

The group highlighted that family caregivers often go through many different situations and deal with various professionals and staff teams throughout their lives. They may find this stressful as they constantly develop relationships with new professionals and staff. This should be acknowledged, particularly if the staff team finds at times the family caregivers challenging.

### Conflict resolution

The group recommended that if there is a conflict between the family caregivers, the staff team, and the multidisciplinary team, a best interests meeting could be held to resolve the issue. Also, an independent advocate could be appointed when necessary and where appropriate. In extreme cases, it may be necessary to use a legal route. For example, in the UK, the Mental Health Act or the Mental Capacity Act may apply or even seek mediation from a court of law. However, it is essential to make every effort to resolve the difference of opinion before any legal help is requested. In an extreme case, the matter could also be referred to the Human Rights Commission, UK Care Quality Commission, or the Clinical Director of Learning Disability in the Department of Health, UK.

The group recommended that an independent professional advocate should look at the facts of the situation or decision to be made and will work with the person with ID/ASD and consult with family caregivers where appropriate. The advocate should be genuinely independent and non-judgemental and be there for the person with ID/ASD to find out what they want or would want if they could communicate this. An advocate could help the person with ID/ASD to speak up and advocate for themselves or speak up on their behalf if necessary. The advocate should not talk negatively about the family caregivers or the person with ID/ASD, even if there are some difficulties with communication and differences in opinion. They should listen carefully to the family caregivers' views and opinions and give them time and a safe space to air their concerns and worries.

The group also recommended that the support staff and independent advocate hold an informal meeting with various stakeholders, including family caregivers and the person with ID/ASD, to discuss views and provide a source of information before a formal best interest meeting/decision. They should think compassionately and creatively about how mutually agreeable compromises could be reached, which is both person and family-centered. They should put themselves in the shoes of the family caregivers to perceive how the family caregivers may be feeling under the circumstances. They should discuss with family caregivers how best to manage BtC and improve the QoL of the person with ID/ASD. It is worth remembering that family caregivers and the person with ID/ASD may feel overwhelmed and daunted to attend a meeting dominated by many professionals. This could be particularly stressful if there is a difference in opinion between the family caregivers and the professionals regarding big decisions such as their move from a house or a significant treatment decision. The group highlighted that Article 8 of the Human Rights Act gives “A right to a private and family life”.

### Train families in STOMP/medication-related issues

It was recommended that the family caregivers be trained and given information about psychotropic medication, when they could and should not be used, their side effects etc. Family caregivers should also be trained on the causes and consequences of BtC and non-pharmacological psychosocial interventions available to address BtC. All these should reduce the overreliance on medication and instead encourage psychosocial interventions for BtC. SPECTROM has two core modules. The Medication module provides information on medication and its indications and side effects. The Alternatives to medication module includes information on how to help people with ID/ASD when they manifest BtC without using medication. Although SPECTROM was primarily developed for support staff, these modules could effectively be used to train family caregivers. The group recommended that training could be provided through videos, e-learning, and online resources from various organizations. They should be given information on monitoring requirements while their relatives are on medication and how to help them at the time of medication withdrawal, including risk management if that is seen is in the best interests of the person with ID/ASD. All these are available through the SPECTROM training.

### Involve family in training

Some training programmes for support staff and other professionals involve people with ID/ASD as trainers. This approach has been a valuable adjunct to any training programme (49, 50). The group recommended involving people with ID/ASD and their family caregivers in training support staff and other professionals where possible and appropriate. This will allow the trainees to hear first-hand from the family caregivers', own experience and views on the causes and effects of BtC and how best to address them.

### Other recommendations

The group recommended that the contact with family caregivers should not be restricted because the person with LD/ASD displays more BtC after family visits or when the person returns from visiting the family home. This could indicate that the person is communicating through BtC that they are not happy to return to the community home. Their needs may be better met elsewhere, or changes needed in the community home and the support offered to the person with ID/ASD. The person with ID/ASD may also struggle with the transition from one setting to another and need to be supported to manage the transition and anxieties around the transition, if necessary, by referring them to appropriate professionals like a speech and language therapist or a clinical psychologist or an occupational therapist or a social worker. Staff should go through the proper channel to discuss with other relevant professionals such as the CLDT members or an independent advocate if they feel an issue of concern with the family visits. Stopping family contacts without going through the proper channels and not following the lawful process could break the law by inappropriately depriving an individual with ID/ASD of their liberty under the Deprivation of Liberty guidance under the Mental Capacity Act in the UK, and also breaching the Human Rights legislation.

The staff team should respect the cultural needs of the person with ID/ASD and their family caregivers. Ensure an interpreter is available if it is necessary for the family caregivers to fully understand what staff are saying to them, particularly in a meeting. Staff need to be mindful about not dragging family caregivers into any internal politics in the community home. It can be very unsettling for a family caregiver to be told about conflicts between staff members that they do not need to be involved with.

If possible and desirable, the staff team should facilitate linking up family caregivers from different families to exchange ideas and information. Similarly, staff can help signpost various services to the family caregivers, such as getting help with finance, accommodation, respite care, etc.

## Discussion

Families are a permanent presence in the life of a person with ID/ASD, whereas support staff and other professionals may come and go. Family caregivers may have vital information about the causes of BtC and its management, which may be unknown to support staff and knowing them helps control BtC. This can reduce the overmedication of people with ID/ASD.

Participants in the co-design day strongly recommended improved information sharing with family caregivers and shared decision-making, mainly to involve them and the person with ID/ASD in deciding on medication use. The group also recommended training family caregivers on the issue of medication use with a particular emphasis on withdrawing medication when appropriate. Currently, family caregivers show a lot of anxiety about medication withdrawal which could be addressed with better training. It was also suggested that family caregivers should also be provided with knowledge that allows early identification of BtC before it reaches a crisis point and be skilled in responding to BtC. Other recommendations included having an open-door policy and enabling family caregivers to visit their relatives without any restrictions, offering privacy during visits, involving independent professional advocates when appropriate, mainly if there is a conflict of interest and identifying a key family caregiver and a key support staff. There was no disagreement among the participants on any of these recommendations, so these recommendations were unanimously agreed upon and consensus-based. Similarly, there was no disagreement in the group on the recommendations made by the independent advocate, who was the only person representing her profession.

These recommendations align with what we found in our previous interviews with family caregivers (28). In our study, the consensus among family caregivers was that they did not have much influence over the decision-making process when it came to care planning for their relatives with ID/ASD. They felt that they did not have enough knowledge about medications and their indications to decide on prescribing for their loved ones. In general, they were keen on alternative approaches to medications to address BtC. This issue is addressed in the current study under the recommendation of training family caregivers on the causes and management of BtC, medication-related issues and alternatives to medication to address BtC. Family caregivers in our previous study (28) seemed to know about the causes of BtC, including communication issues, underlying mental health and environmental factors. The family caregivers wanted staff and professionals to listen to them and involve them in decision-making. The current study came up with similar recommendations. In our previous study (28), family caregivers wanted more support for the support staff themselves, which led us to develop the SPECTROM training programme. In the current study and our previous study (28), family caregivers expressed concern about the possible side effects of medication withdrawal, particularly in the form of deterioration in the person's behavior.

In another study involving interviews with family caregivers, Sheehan and colleagues (27) found that some family caregivers were involved in the decision-making process involving their loved ones, particularly those whose children were younger than 18, but others, mainly whose relatives are adults, did not feel so involved. This view was endorsed by the family caregivers and the independent advocate in the co-design event, who stated that they were often not involved in decision-making and were only informed about decisions after they were made. Views of the family caregivers on the use of medication to address BtC in Sheehan's et al. study (27) were divided. Some felt medication is necessary, but others felt that often there is an over-reliance on medication when it comes to managing BtC rather than exploring all interventions, particularly psychosocial/behavioral, and taking a holistic approach to this. A similar division in opinion was observed in the focus group, where some support staff felt the use of medication was justified, whereas others felt this was a “chemical restraint” (51). Family caregivers have inconsistent knowledge and perception of the use of psychotropic medications for BtC. This could be due to the lack of information, as stated by some participants in the current study. Similar to the current study, family caregivers in Sheehan's et al. (27) study wanted more information on the medication and expressed concern about the medication's side effects. For this concern, in the SPECTROM module, we have recommended staff to provide information and resources that will encourage family carers to train themselves on topics of interest, such as psychotropic medications and their side effects, BtC and management strategies and so on.

Like the current study, previous studies highlighted the issues of a lack of partnership working and neglect of family caregivers' knowledge and opinion (26, 34). Family caregivers often felt it was a “battle” to be recognized and access support and lacked clear and understandable information about their relatives' care (21, 26, 52–55).

The co-design day was a success but required a lot of preparation beforehand to plan the event. Every participant was engaged with the activities, and the co-design event appeared well-paced. We adapted the EBCD as the original phases of EBCD suggested by the toolkit could not be accommodated within the project timescale. For example, we could not carry out some of the methods of capturing experiences as suggested by EBCD, such as observing or shadowing participants or filming interviews to understand the experiences of service users or staff etc., as they were time-consuming and resource-intensive. SPECTROM used focus groups, meetings and workshops, teleconferences, synthesis of existing evidence and a one-day workshop to capture the experiences of participants and suggestions for its contents and the format. The SPECTROM programme was successfully developed after many revisions (https://spectrom.wixsite.com/project) (39).

Our study shows that the EBCD approach can be successfully adapted and used to develop a training programme. Other interventions have also been developed using this method but in different fields and targeting various health care services (56, 57). Developing an intervention while keeping stakeholders and service users at the heart of the development process will help make the intervention more relevant and practical for the audience and the purpose of the intervention. This helps to provide face validity of the intervention. Our study should encourage others to use the EBCD approach to develop training programmes collaboratively.

### Strengths

The co-design event used a modified version of evidence-based method called EBCD to gather stakeholders' equal input and experiences from the beginning of the project. The co-design event findings that helped develop the module “Effective liaison with family carers and advocates” were based on the real experiences of stakeholders, which were used as evidence. Hence, the module was developed using evidence-based method. All recommendations and action plans from the co-design event were clearly documented. It showed the outline of what information should be included in each module, what format this should take, and how to deliver it. Thus, the co-design event that helped develop SPECRTOM is replicable and auditable. Another strength is that there was no researcher bias as the participants worked on the activities independently, ensuring no influence from the researchers.

### Limitations

One limitation was that we could not use EBCD as suggested by the toolkit, and it had to be modified to fit our project timescale. For example, SPECTROM was unable to utilize videos to help capture experiences. However, we still had a successful and meaningful co-design event without it, where detailed information was gathered. Another weakness is that EBCD is resource-intensive, and future projects need to ensure that they have enough resources to carry out EBCD successfully. On the day of the co-design event, five participants did not attend. Hence, not all tables/groups had all stakeholders' groups represented to provide their input to the theme/topic. The other drawback was the lack of direct participation of people with ID in the event, although their input was captured more effectively through their advisory group.

## Data availability statement

The raw data supporting the conclusions of this article will be made available by the authors as long as permitted by the funder and the sponsor of the project and if there are no other legal restrictions, without undue reservation.

## Author contributions

SD is the grant holder. Both authors were involved in the conception and design of the study, planning, and holding the co-design event. BL analyzed the co-design data. Both authors contributed substantially to the preparation of the manuscript and approved the final version of the manuscript.

## Funding

This study was funded by the UK's National Institute of Health Research (NIHR) Research for Patient Benefit (RfPB) Programme (Grant No. PBPG-0817-20010). The Imperial Biomedical Research Center Facility, which is funded by the NIHR, has provided support for the study.

## Conflict of interest

The authors declare that the research was conducted in the absence of any commercial or financial relationships that could be construed as a potential conflict of interest.

## Publisher's note

All claims expressed in this article are solely those of the authors and do not necessarily represent those of their affiliated organizations, or those of the publisher, the editors and the reviewers. Any product that may be evaluated in this article, or claim that may be made by its manufacturer, is not guaranteed or endorsed by the publisher.

## Author disclaimer

The views expressed in this article are those of the authors and not necessarily those of the National Health Service, the NIHR, or the Department of Health, UK.
