# Supplementary material for: Support staff liaising effectively with family caregivers: Findings from a co-design event and recommendation for a staff training resource
Source: Front Psychiatry. 2022 Sep 14;13:977442. doi: 10.3389/fpsyt.2022.977442 (PMC9555056; doi:10.3389/fpsyt.2022.977442)
Supplement: Supplementary file 1 [file Table_1.docx]

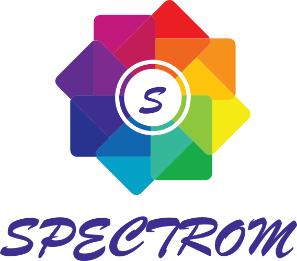
Group 2

We need your help to develop the modules for SPECTROM Psychoeducational Programme (PEP). You are given one hour to complete all these tasks. Do not worry if you cannot complete all tasks, but please ensure you complete task 1 in one hour. If time allows, you can try to complete other tasks in the order they are written.

Here is a guide (but not a prescription) for time management; (a) first 15 minutes: introduction and brain storming/ general discussion; (b) 30 minutes: content text/ case vignettes; (c) 10 minutes: rest of the tasks (if time allows); (d) 5 minutes: summarise main points/ scribe on flip chart for presentation.

Please use the paper to scribe the main points from your discussion. Please select a spokesperson from the group who will present the main findings to the whole group when you finished your task. Please write a more detailed script, if possible (over and above the main summary points for the presentation), which you could either hand over to Bharati at the end of the session or email us later.

You will have five minutes to do the presentation, allowing for one or two questions/clarifications from the floor.

There will be scope for further discussions at the end of the afternoon task session.

The PEP will have a written module and a face-to-face training session primarily geared towards service managers, senior care staff and those care staff who deal with severe challenging behaviour. The written modules could be accessed by family carers or CLDT members, or any other stakeholders.

Therefore, please keep this audience in mind when preparing the contents of your theme. Therefore, we propose to produce basic modules and more detailed/specific areas of modules to cater for different levels of knowledge and expertise.

Task 1

Your group is allocated the following theme.

**Effective liaison with family carers/advocates**

We would like you to consider the contents that need to be included in this PEP module (what information will the care staff team need to effectively liaise with family carers and advocates). Please feel free to add texts associated with each content if possible. Please also think of case vignettes to accompany appropriate content/text.

Some examples of the contents are;

*“How to effectively communicate with family carers and advocates?”*

*“How to maintain communication between care staff and family carers/ advocates?”*

*“When to inform and involve family carers and advocates in decisions and procedures?”*

*“How to address a difference in opinion between care staff and the family carers?”*

These are what some of our participants in the Focus Group said about the difficulty in liaising with family carers and advocates:

Trainer

“More often, when family is really quite involved with the service user, so, therefore, will have them home for the weekend or have them for longer periods of time. The fear of the change in medication will have an effect on them and how much more support they’re going to have to give them or how their behaviours might become more challenging when they have them at the weekend. In a different environment which again can change behaviour as well.”

Service Manager

“Sometimes there’s a parent who is going to tell you I know my child and I think they need this, they have been using this. Where the family, if that’s the next of kin, you have what you are saying ready, but the decision comes from someone else. Sometimes parents are very resistant where they’re going.”

Trainer

“Just to share one experience with you where somebody we're supporting was being withdrawn from the medication and everybody had bought into it, but when they went home for the weekend the family still had that medication there and they will administer it, because they had their own supply at home.”

Task 2

Once you have developed the main contents/texts/case vignettes, please think and describe how the contents of the module could be implemented in a day to day practice.

Task 3

Please rank the contents you have produced in terms of their hierarchy from basic to specific and detailed. So that we can decide what to include in a basic module and also in more specialised modules.

Task 4

Please think and describe how the written modules could be linked to the face-to-face training session (e.g., the trainer to explain how to use the written module in the context of a real-life scenario, how to assimilate information from the written module, and answer any question related to the module).

Task 5

If you have spare time, please feel free to contribute to the contents/case vignettes for other themes that were not originally allocated to you.
