# Supplementary material for: Support staff liaising effectively with family caregivers: Findings from a co-design event and recommendation for a staff training resource
Source: Front Psychiatry. 2022 Sep 14;13:977442. doi: 10.3389/fpsyt.2022.977442 (PMC9555056; doi:10.3389/fpsyt.2022.977442)
Supplement: Supplementary file 2 [file Table_2.docx]

**Challenging behaviours: Views and preferences of people with intellectual disabilities**. *JARID 2019, Dutch study*.

**Feels negative about the following, which is likely to precipitate challenging behaviour (CB).**

1. Lack of structure and clarity in daily life.
2. Unsure what care staff and others want of them.
3. Care staff and others putting too much demand on them.
4. Lack of other’s understanding of expressive vs. comprehensive speech discrepancy.
5. Inability to cope with own emotion, feeling and loneliness.
6. Misunderstood or ignored.
7. Negative events in life.
8. Some agree to take medicine but most want to discontinue.
9. A psychiatric diagnosis helps to clarify things for some, but others find it difficult to accept.
10. Care staff are angry and confrontational.

**Feels positive about the following, which is likely to improve challenging behaviour (CB).**

1. More clarity about the structure of daily routine from care staff and others.
2. Clear understanding of what care staff and others want from them.
3. Understanding of communication issues (e.g., behaviour is a means of communication; expressive vs. comprehensive speech discrepancy; impaired social communication as in ASD; attention deficit affecting communication in ADHD; different/simplified psychological defence mechanism/coping strategies).
4. Psychoeducation to themselves and care staff (to gain better understanding of the issues).
5. Early detection plan and a clear plan to address them (include all stakeholders).
6. Give their own opinion on the intervention strategy (including the use of medication).
7. Doing physical exercise, playing sports.
8. EMDR.
9. Group therapy.
10. Assertiveness course.
11. Get on with care staff.
12. Better understanding of the person’s capabilities to avoid too much demand and collaborative approach to work/daily routine plan.
13. Explain psychiatric diagnosis (if one is made) clearly & thoroughly (take time, make reasonable adjustment, use pictures and other accessible formats).
14. More information about pharmacological intervention.
15. Help with discontinuation of inappropriate medicine use.
16. Care staff characteristics (calm, trustworthy, honest, friendly, engaging, empathetic etc.)

**How service-users with intellectual disabilities understand challenging behaviour and approaches to managing it?** *Clarke, Dagnan, Smith 2019, English study*.

1. **Internal frame of reference** (*recognition of own feelings, stress, thought, behaviour*)
2. Listening to music.
3. Go to her own room.
4. Relaxation exercise etc.
5. Reflective conversation with care staff they trust or in a group about an incident.
6. ‘insight-oriented intervention’.
7. (more able they feel about controlling own stress and behaviour and in control leads to a better positive cycle of confidence to deal with these in future so reducing CB).
8. **External frame of reference** (*environmental issues*) (*lack of internal locus of control*)
9. Opportunities for skill building.
10. ‘Active Support Framework’.
11. Care staff training people with intellectual disabilities in conflict resolution, structured problem solving in group formats.
12. CAT, CBT, narrative therapy.
13. Involvement of the person with intellectual disabilities in functional assessment to help with the shared understanding.
14. **Positive relationship** (*with peers + care staff + family members*) (get on with some but not others, may lead to stress & CB)
15. Care staff to teach the person with intellectual disabilities anger management.
16. Care staff to teach conflict resolution, feel/think/behave in a positive alternative way.
17. Care staff to show more empathy and not to take things personally.
18. (family support provides a sense of belonging).
19. **Exert power and control in day to day life** (*power & trust from service* + *power & choice/independence to do things*)
20. Risk management vs. trust/independence to carry out tasks.
21. Care staff working collaboratively with the person with intellectual disabilities.
22. Go slow.
23. Develop skills over time.
24. Monitor progress.
25. Provide timely support.
26. Revise strategy/use alternative skill building if/when necessary.
