# Supplementary figures and images for: Support staff liaising effectively with family caregivers: Findings from a co-design event and recommendation for a staff training resource
Source: Front Psychiatry. 2022 Sep 14;13:977442. doi: 10.3389/fpsyt.2022.977442 (PMC9555056; doi:10.3389/fpsyt.2022.977442)

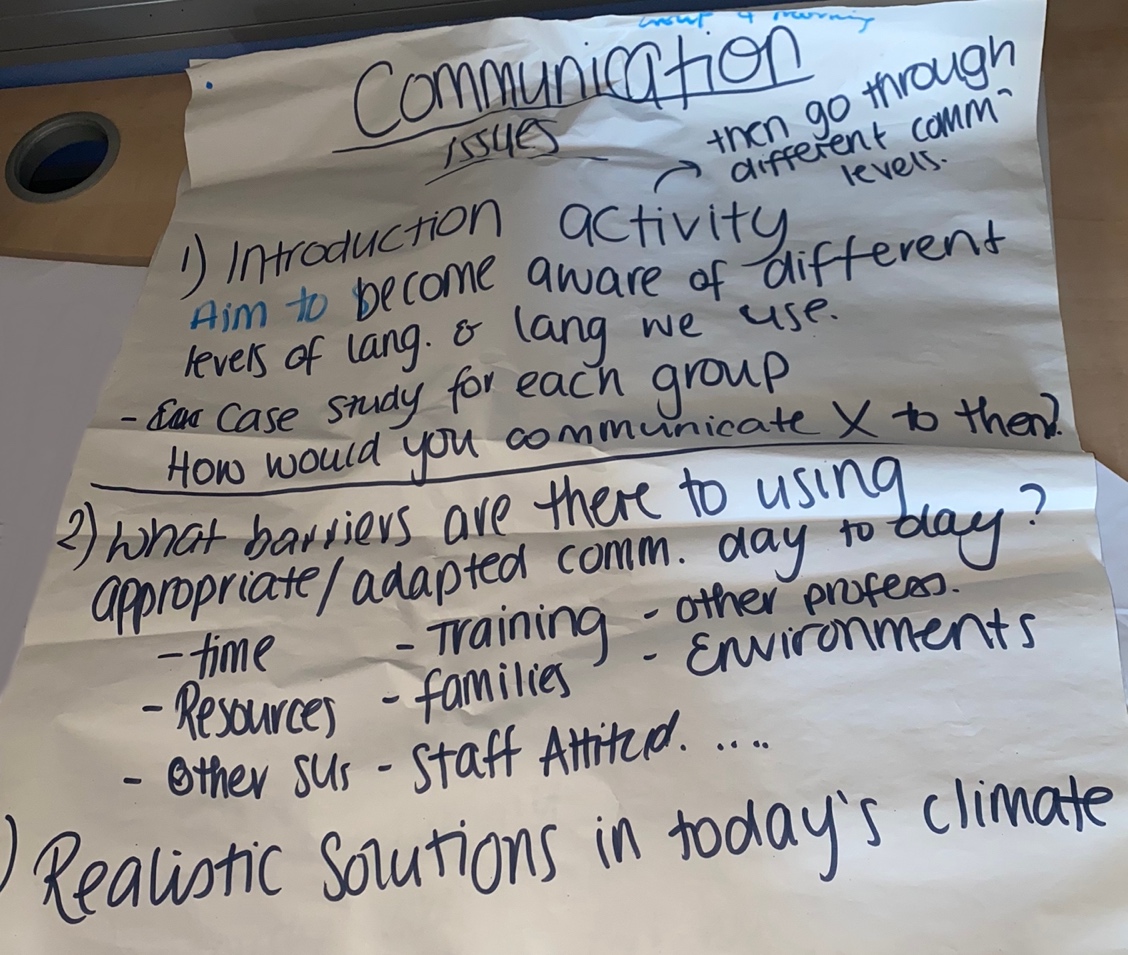


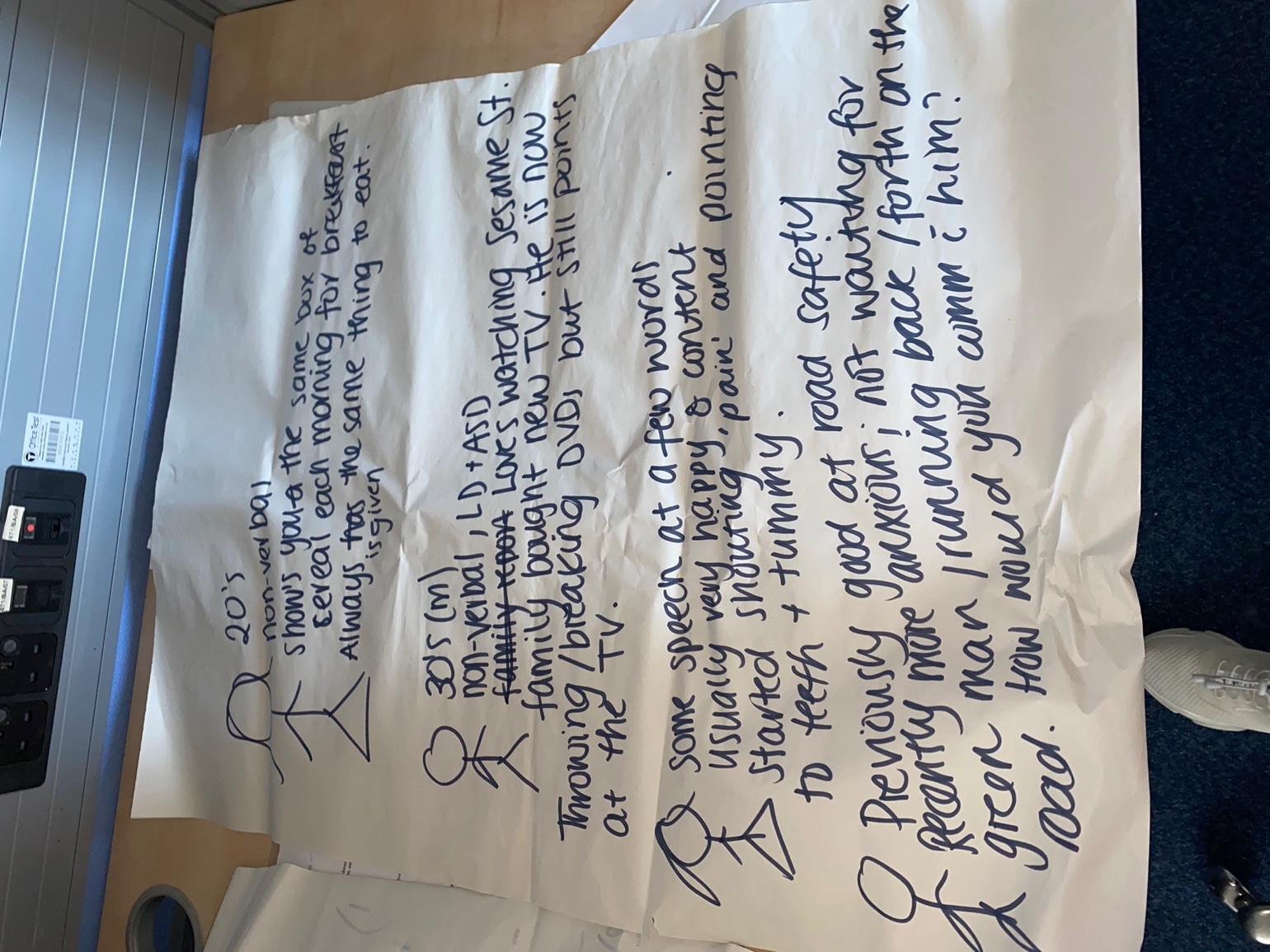

Supplement: Supplementary file 3 [file Table_3.docx]
